# Monitoring *Aspergillus flavus* Genotypes in a Multi-Genotype Aflatoxin Biocontrol Product With Quantitative Pyrosequencing

**DOI:** 10.3389/fmicb.2019.02529

**Published:** 2019-11-15

**Authors:** Kenneth C. Shenge, Bishwo N. Adhikari, Adebowale Akande, Kenneth A. Callicott, Joseph Atehnkeng, Alejandro Ortega-Beltran, P. Lava Kumar, Ranajit Bandyopadhyay, Peter J. Cotty

**Affiliations:** ^1^United States Department of Agriculture, Agricultural Research Service, Tucson, AZ, United States; ^2^International Institute of Tropical Agriculture, Abuja, Nigeria; ^3^International Institute of Tropical Agriculture, Ibadan, Nigeria

**Keywords:** pyrosequencing, aflatoxin, Aflasafe, biocontrol, atoxigenic, monitoring, maize, Nigeria

## Abstract

Aflatoxins pose significant food security and public health risks, decrease productivity and profitability of animal industries, and hamper trade. To minimize aflatoxin contamination in several crops, a biocontrol technology based on atoxigenic strains of *Aspergillus flavus* is commercially used in the United States and some African countries. Significant efforts are underway to popularize the use of biocontrol in Africa by various means including incentives. The purpose of this study was to develop quantitative pyrosequencing assays for rapid, simultaneous quantification of proportions of four *A. flavus* biocontrol genotypes within complex populations of *A. flavus* associated with maize crops in Nigeria to facilitate payment of farmer incentives for Aflasafe (a biocontrol product) use. Protocols were developed to confirm use of Aflasafe by small scale farmers in Nigeria. Nested PCR amplifications followed by sequence by synthesis pyrosequencing assays were required to quantify frequencies of the active ingredients and, in so doing, confirm successful use of biocontrol by participating farmers. The entire verification process could be completed in 3–4 days proving a savings over other monitoring methods in both time and costs and providing data in a time frame that could work with the commercial agriculture scheme. Quantitative pyrosequencing assays represent a reliable tool for rapid detection, quantification, and monitoring of multiple *A. flavus* genotypes within complex fungal communities, satisfying the requirements of the regulatory community and crop end-users that wish to determine which purchased crops were treated with the biocontrol product. Techniques developed in the current study can be modified for monitoring other crop-associated fungi.

## Introduction

Several species of *Aspergillus* section *Flavi* produce immunosuppressive, hepatotoxic and carcinogenic aflatoxins (Liu and Wu, 2010; Liu et al., 2012) in maize and other crops cultivated in warm environments (Cotty et al., 1994; Cotty and Jaime-Garcia, 2007; Bandyopadhyay et al., 2016). At high concentrations, aflatoxins may cause acute hepatotoxicity, hemorrhagic liver necrosis, and death (Probst et al., 2007, 2010, 2011). For this reason, levels of aflatoxins in foods and feeds are strictly regulated in more than 100 countries across the world (FAO, 2004; EU, 2010; Matumba et al., 2017; Singh and Cotty, 2017), and aflatoxin management strategies, including biological control, are used for mitigating aflatoxin exposure. Commercial biological control products directed at aflatoxin mitigation have beneficial strains of *A. flavus* that do not produce aflatoxins as active ingredients.

*Aspergillus flavus* consists of many genetically distinct groups, called vegetative compatibility groups (VCGs), that primarily reproduce clonally (Grubisha and Cotty, 2010, 2015; Ortega-Beltran et al., 2016; Islam et al., 2018) and differ widely in several characteristics, including ability to produce aflatoxins. Aflatoxin-producing potential varies more between VCGs than within them (Bayman and Cotty, 1991); all members of certain VCGs lack the capacity to produce aflatoxins (Cotty et al., 2008; Grubisha and Cotty, 2015; Ortega-Beltran et al., 2016) and are referred to as atoxigenic. Adoption of biocontrol strategies utilizing indigenous atoxigenic genotypes to displace aflatoxin producers in crop-associated fungal communities (Atehnkeng et al., 2008a,b; Mehl et al., 2012) are becoming widespread across the world, due to proven efficacy, low cost, and area-wide benefits (Cotty and Bayman, 1993; Cotty and Bhatnagar, 1994; Dorner, 2008; Atehnkeng et al., 2014; Mauro et al., 2015; Bandyopadhyay et al., 2016). This strategy alters compositions of crop-associated fungal communities through founder effects, competitive displacement and other mechanisms (Cotty and Mellon, 2006; Ortega-Beltran and Cotty, 2018), increasing frequencies of atoxigenic active ingredients and decreasing incidences of aflatoxin-producers through displacement (Abbas et al., 2011; Chang et al., 2012; Atehnkeng et al., 2014). However, after application, monitoring active ingredient genotypes in the *A. flavus* community is necessary to assess influences of various practices on displacement of aflatoxin producers by applied atoxigenics. Monitoring is also required to verify use of the biocontrol products on crops where use is rewarded, as with farmers supplying crops under the Nigeria Aflasafe™ Challenge Project (AgResults, 2019; Schreurs et al., 2019).

As part of post-application monitoring, vegetative compatibility analyses (VCA) are frequently performed to determine displacement efficacy and residual effects (Cotty and Bayman, 1993; Atehnkeng et al., 2014; Mauro et al., 2015). VCA involves generation of nitrate non-utilizing auxotrophs for individual isolates, pairing of auxotrophs with *cnx*^−^ and *niaD* tester pairs, and classification of the complementing fungi as a member of the VCG defined by the tester pair (Bayman and Cotty, 1991; Grubisha and Cotty, 2010). A test must be performed for each isolate and limitations on the assays are imposed by both the isolation process and the number of isolates that can practically be classified. This process is laborious, expensive, and time-consuming, frequently talking over a month to complete. Pyrosequencing assays can reduce costs and increase speed and accuracy of post-application biocontrol monitoring, quantitative pyrosequencing assays targeting specific *A. flavus* isolates have been developed (Das et al., 2008; Mehl and Cotty, 2010, 2013). However, none of these have been successful at monitoring multiple genotypes, and none have been used to monitor commercially significant quantities of samples.

The current study aimed to develop multi-genotype quantitative pyrosequencing assays for quantification of *A. flavus* genotypes. This was predicated on previous success with single-genotype assays (Das et al., 2008; Mehl and Cotty, 2011, 2013) and the need for rapid verification of biocontrol-use on maize. Aflasafe is a commercially available biocontrol product with four endemic *A. flavus* genotypes (as active biological ingredients) isolated from Nigeria for reducing aflatoxin contamination in maize (Atehnkeng et al., 2008a,b, 2014; Bandyopadhyay et al., 2016). Proper use of this biocontrol in fields before flowering leads to occurrence of significant frequency of the active biological ingredients. This initiative, a project under the AgResults multilateral initiative (AgResults, 2019) aims to provide incentives for aflatoxin-mitigation through increased adoption of biocontrol through a performance payment per unit of maize (~30 tons) that is verified to contain significant frequencies of the active ingredients. The active ingredients must be detected rapidly and precisely to enable accurate and timely implementation of one of the project objectives for paying the incentive. The current work describes efforts to meet these needs with pyrosequencing.

Quantitative pyrosequencing is an advanced sequence-based technology that enables accurate quantification of frequencies of DNA sequence variants in complex microbial populations. Pyrosequencing relies on light generation after nucleotides are incorporated in a growing DNA strand, converting the emitted light into a pyrogram. Pyrogram peaks correspond to light generation, and is proportional to nucleotide incorporation (Siqueira et al., 2012). Pyrosequencing produces a large number of sequence reads in a single run, resulting in enormous sampling depth (number of sequences per sample) that permits detection of both dominant and rare individuals within mixed and complex microbial populations by several orders of magnitude higher than previous technologies allowed (Sogin et al., 2006; Kunin et al., 2010; Mehl and Cotty, 2010). Greater sampling makes pyrosequencing especially suitable for ecological studies, such as monitoring changes in *A. flavus* population structure (Das et al., 2008; Mehl et al., 2012) or incidences of single nucleotide polymorphism (SNP)-based fungicide resistance (Zhou and Mehl, 2019). The current study developed quantitative pyrosequencing assays for quantifying frequencies of active ingredients of Aflasafe in complex microbial populations. The biocontrol product consists of equal proportions of four atoxigenic *A. flavus* isolates (Ka16127, La3279, La3304, and Og0222) (Atehnkeng et al., 2014). Assays were based on SNPs in the genomes of each genotype, and were for specific detection of the target genotype. Multi-isolate assays targeting more than one active ingredient were based on SNPs shared by the target genotypes.

## Materials and Methods

### Detection of Single Nucleotide Polymorphisms in Target *Aspergillus flavus* Genomes

Sequenced reads from the target isolates (Ka16127, La3279, La3304, and Og0222), and control isolates (AF13, MS14-19, and Ss19-14) were mapped to *A. oryzae* RIB40 genome (Machida et al., 2005) using Bowtie v1.1.1 (Langmead et al., 2009). The resulting BAM files were used as input to SAMTools v0.1.16 (Li et al., 2009). SNP positions were identified using mpileup function in SAMTools. SNPs with minimum mapping quality (-Q) below 20 and minimum read coverage below 20 × were filtered out. SNPs specific to one isolate, or shared by multiple isolates, were identified using a custom Perl script, and polymorphic regions were validated by checking alignment of the target and control isolates with the reference. Regions containing putative SNPs were annotated using BLAST (Altschul et al., 1990) against non-redundant databases. Target genomic regions with polymorphisms were aligned with sequence from other *A. flavus* isolates reported previously (Adhikari et al., 2016) to ensure that target genomic regions were highly conserved, increasing the potential that allele quantification with the pyrosequencing assays would more accurately reflect the diversity in fungal populations within the communities being assayed. Further checks were done to ensure that target SNPs were not within genes located in the aflatoxin biosynthesis cluster. Since many biocontrol *A. flavus* isolates have lost all or part of the aflatoxin biosynthesis cluster (Adhikari et al., 2016) this genomic region would not be a suitable target for *A. flavus* population studies.

### Extraction and Amplification of Target DNA

Total DNA was extracted from maize grain by suspending 10 g of ground maize in 50 ml of 0.1% TWEEN®80. After shaking for 20 min at 175 rpm, the suspension was transferred into a funnel lined with a 4 × 4 in piece of Miracloth (EMD Millipore, Billerica, MA) and vacuum-filtered into a 50 ml centrifuge tube. The flour residue was autoclaved and discarded. After centrifuging the filtrate for 10 min at 4,000 × g, the supernatant was removed, using a 10 ml serological pipette, and discarded. The precipitate was vortexed at 15,000 rpm for 15 s, after which 1 ml, containing maize starch and fungal propagules, was transferred to a 1.5 ml microfuge tube and centrifuged at 8,000 × g for 5 min. The supernatant was again removed, using a 1,000 μl pipette, 450 μl of Lysis Buffer (30 mM Tris, 10 mM EDTA, 1% SDS, pH 8.0) was added, and the tube was vortexed to suspend the precipitate. The tube was then placed in a Thermomixer for 60 min at 60°C and 8,000 rpm, after which it was centrifuged at 14,000 × g for 30 min, and 370 μl of the supernatant transferred to a new 1.5 μl microfuge tube to which 370 μl of 4 M ammonium acetate (NH_4_OAc) was also added. After mixing the suspension by inverting it several times, 740 μl of ice-cold ethanol was added. The microfuge tube was incubated at −20°C for 30 min, centrifuged at 14,000 × g for 5 min, the supernatant was removed, and the DNA pellet dried by placing the tube upside-down on a paper towel for about 60 min. The DNA was re-suspended in 25 μl of sterile water and quantified using a NanoDrop™ ND-3300 Fluorospectrometer (NanoDrop Technologies, Inc., Bancroft, DE).

Target *A. flavus* DNA was amplified using nested PCR (Dufour, 1977; Sun et al., 2012), with sequential DNA amplifications. The first amplified a relatively large section (400–580 bp) surrounding the SNP. Outer primers were designed using Primer3Plus (http://www.bioinformatics.nl/cgi-bin/primer3plus/primer3plus.cgi). Inner primers, were designed using PyroMark Assay Design Software v2.0.1.15 (Qiagen, Valencia, CA) to amplify 90–180 bp containing the target SNP, within the larger outer amplicon. Inner primers were purified using high-performance liquid chromatography (HPLC), and either the forward or reverse primer was tagged with biotin at the 5′ end for biotinylation of the inner PCR amplicon. Biotinylation allows subsequent attachment of the amplicon to Streptavidin Sepharose beads during the pyrosequencing reaction. PCR amplifications used *AccuPower* Hotstart PCR PreMix tubes (Bioneer, Inc., Alameda, CA). Each tube contained a pre-mix of one unit of HotStart DNA polymerase, 1 × PCR Buffer and 250 μM of each dNTP. The 20 μl reaction mixture in each tube included 0.5 μl each of the forward and reverse primer, 17 μl of deionized water and 2.0 μl of the DNA template, diluted to 5 ng/μl concentration. Amplicons from the outer reaction served as templates for inner PCR. Amplification conditions were DNA denaturation (94°C, 5 min) followed by 38 cycles of melting at 94°C for 20 s, primer annealing at 56°C for 30 s, extension at 72°C for 30 s, and a final extension step at 72°C for 10 min. Amplicons were visualized with GelRed (Biotium Inc., Fremont, CA), using a G:Box Chemi HR 16 Bio Imaging System (Syngene/Synoptics Ltd., Cambridge, UK), after separation on 1.0% agarose gel via electrophoresis at 110 volts for 15 min. Proportions of reagents in the reaction mix and amplification conditions were the same for outer and inner PCR amplifications.

Outer amplicons were quantified using a Qubit 3.0 Fluorimeter (Thermo Fisher Scientific, Waltham, MA) for initial standardization of the PCR protocol. Serially diluted amplicons, up to 10^−3^, were used as DNA template for inner reactions to determine optimal dilutions. Total DNA extracted from ground maize was a mixture of DNA from *A. flavus*, maize, and environmental organisms. Therefore, the amplicon size and brightness on the gel was used as a guide to determine the quantity of PCR products to be used as template for the inner PCR reaction.

### Confirmation of Predicted SNPs

Design of outer primers for quantitative pyrosequencing assays was based on SNP prediction by computational analyses. Actual presence of the predicted SNPs was confirmed by sequencing amplicons at the University of Arizona Genetics Core (UAGC) facility, using Applied Biosystems 3730XL DNA Analyzer (Life Technologies Corporation, Carlsbad, CA). Sequence data were aligned to reference genomes using Geneious® 9.0.2 (Kearse et al., 2012). Design of inner and sequencing primers followed SNP confirmation.

### Quantitative Pyrosequencing Assays

Quantitative pyrosequencing assays were designed using PyroMark Assay Design Software v2.0.1.15 (Qiagen, Valencia, CA) and performed on a PyroMark Q24 Pyrosequencer (Qiagen, Valencia, CA). Twenty-four assays were developed (Table 1), consisting of forward and reverse pairs of outer and inner primers and a sequencing primer (Table 2). Six of the assays (1Ka1–1Ka6) were for Ka16127, six were for La3279 (1La791–1La796), four were for La3304 (1La041–1La044) and two were for Og0222 (1Og1 and 1Og2) (Tables 1, 2). Two assays (2KaLa2 and 2La9K2) were designed for simultaneous quantification of Ka16127+La3304 and La3279 + Ka16127, respectively, while the remaining six assays (3La94K1–3La94K6) were for simultaneous quantification of Ka16127 + La3279 + La3304. Template DNA preparation for pyrosequencing analysis was done following procedures described by Das et al. (2008).

**Table 1:** Targets for differentiating atoxigenic *Aspergillus flavus* active ingredients of Aflasafe identified with whole genome analyses and confirmed with amplicon sequencing.

**Assay^#^**	Target *A. flavus* isolate^*^	**Variable Sequence (Bold letter = SNP)**	**SNP**	**IUPAC ambiguity code**	**Amino acid change**	**Location on *A. oryzae* genome^**#**^**	**Polymorphic site annotation**
1Ka1	1	TTCCGGTAT**G**TGCAAAGCGG	A → G	R	Y → C	Chr. 1, SC009	Polyketide synthase
1Ka2	1	TAGCGATTG**C**GCGGCCCCGC	T → C	Y	V → A	Chr. 5, SC113	DNA repair protein Nse1
1Ka3	1	TCGTTCAAT**A**CAATCAAGTA	G → A	R	C → Y	Chr. 5, SC113	Hypothetical protein AOR_1_1238094
1Ka4	1	GCCTGCCTA**T**TTGCCAATGA	C → T	Y	C → T	Chr. 5, SC113	Haloacid dehalogenase
1La791	2	CGTTACATG**C**GAATCAATAA	G → C	S	G → R	Chr. 1, SC009	Polyamine transporter 3
1La792	2	TTGGCAAGC**A**CCGGCGGAGC	G → A	R	R → H	Chr. 2, SC003	Unnamed protein product
1La793	2	AGCCACTTG**T**TCGATCTTCT	C → T	Y	C → Y	Chr. 1, SC009	Unnamed protein product
1La794	2	AGGGCCCCA**C**GACCAGCATA	A → C	M	R → R	Chr. 1, SC009	Hypothetical protein
1La795	2	GGCTGGACG**T**TTCGGCAACC	C → T	Y	A → V	Chr. 1, SC009	Unnamed protein product
1La796	2	TGTGGAGTT**T**ATGTTTCGTC	C → T	Y	S → L	Chr. 1, SC009	3-ketosteroid-delta-1-dehydrogenase
1La041	3	TTACTGGTG**T**GATCGCTGCG	G → T	K	L → L	Chr. 6, SC020	Guanine nucleotide exchange factor
1La042	3	GGTGGACCA**T**ACGGGATGAA	C → T	Y	H → Y	Chr. 6, SC020	No significant similarity found
1La043	3	GACGCCACC**T**GGTCTCCAGG	C → T	Y	P → L	Chr. 6, SC020	20S cyclosome subunit (APC1/BimE),
1La044	3	GCAGGCACT**C**AAATCTCACC	T → C	Y	^*^ → Q	Chr. 6, SC020	Putative anucleate primary sterigmata (ApsB)
1Og1	4	CAATACCCG**C**ATTATCTTCA	T → C	Y	Y → H	Chr. 3, SC023	Unnamed protein product
1Og2	4	GCCCAAGTG**G**TTCTGGCTAC	C → G	S	C → W	Chr. 3, SC023	Fungal alpha-L-arabinofuranosidase
2KaLa2	1 + 3	ACAACACGG**G**CTTCCAGGAG	A → G	R	G → G	Chr. 5, SC113	Hypothetical protein AFLA70_215g002270
2La9K2	1 + 2	AGCCGGGTC**C**TCCTCTGTGT	A → C	M	H → P	Chr. 8, SC010	Acetylcholinesterase
3La94K1	1 + 2 + 3	TGACTCGAC**T**ATCTTGCTTA	C → T	Y	G → Y	Chr. 8, SC010	Endo-1,4-beta-mannosidase
3La94K2	1 + 2 + 3	CAGGAGCGC**G**TCTCTAAGCT	A → G	R	H → R	Chr. 8, SC010	AMP-binding enzyme
3La94K3	1 + 2 + 3	AAAACGGGC**A**GCATGATGAT	G → A	R	P → K	Chr. 6, SC020	Unnamed protein product
3La94K4	1 + 2 + 3	ACGGCCGAA**C**GAGTCGCTCG	T → C	Y	V → P	Chr. 6, SC020	Unnamed protein product
3La94K5	1 + 2 + 3	TGGCTACTC**T**AAGGTTCTCG	C → T	Y	S → S	Chr. 6, SC020	Hypothetical protein Z518_10119
3La94K6	1 + 2 + 3	AAAAGCGGT**G**CCAAAGGCG	A → G	R	Y → C	Chr. 6, SC020	Hypothetical protein AFLA_104000

# *Numbers preceding letters in Assay names indicate the number of A. flavus isolates targeted by the assays. Where there is more than one assay per A. flavus isolate, letters in the assay name are followed by the serial number of the assay*.

* *1 = Ka16127, 2 = La3279, 3 = La3304, 4 = Og0222, 1 + 2 = Ka16127 + La3279, 1 + 3 = Ka16127 + La3304, 1 + 2 + 3 = Ka16127 + La3279 + La3304*.

**Table 2:** Oligonucleotide primer sets for quantitative pyrosequencing assays directed at polymorphisms described in Table 1 for estimation of frequencies of *Aflasafe* active ingredients in fungal communities associated with maize produced in Nigeria.

**Assay name^#^**	**Outer primers**	**Inner primers**	**Sequencing primer**	Target biocontrol isolate^*^	**Amplicon size (bp)^§^**
1La041	tctgctgcgtacctcattcg/aggctctgaattgcgaacga	CTCAAGCTCGACGTGGCTTAC/ACGGTAGAGGTCAGGTTCTGC	GCCGGCGCAGCGATC	3	514/188
1La042	ACCACCCATATTTAGCGCATCCT/CGAAGCGCGCAGTTGTTAGC	CCTATCGTCGACCATTTAAGGTAA/AAATCCCTAGCCAAAGACGC	GGCACGTTCATCCCG	3	539/169
1La043	CATCGTGTGGCCTTCGACGC/TTTTCGAGGACCAGCGCGC	TTCAAAAGCAGAGACTCCCACTTC/CTGCGCAAACCACTCGGA	GGACAATAAATGGTTCGAT	3	511/103
1La044	AAGGAGGAGGCGCGGAAACT/CAGTCCGGTCCACACATCGC	TGCGCTACTTGAGAGCCACG/CCGAGATGCTTGGTGGTGAG	CGTTTGAGCAGGCAC	3	542/130
1Ka1	GAGCTGTGATCTACGCGACA/ACAAGAAGGTACGACGCGTT	GGTCCCATCAACCCAGTTAC/GATAATCTTCCCCATGTGCTG	GTGCTGTCCGCTTTG	1	509/93
1Ka2	CAGTCACGGTTACCACAACG/TTCCTTTATCAAGCGCATCC	TACCGTTTCCGCTTGAGACAT/ATCGTCCGGAGATGCAAGT	GAGACATGCTTAGCGA	1	463/97
1Ka3	AGTCAGTGGGTCGAAAAAGG/ACAGCGAAGGTTTGACTGCT	TTCATGTTAACGACATCCGTGATC/GGTGGGACAGTTCTTCATGTTGC	GCTGCCAGATACTTGATT	1	414/98
1Ka4	AACAACAGGTGCCAAGTGTG/CTTTGCATTTGCCGGATAAC	TTCGCCAAGAGTGCTCCT/GATCCCATTTAGCCTATGTCTGAG	GGGCGGTCATTGGCA	1	482/96
1Og1	GTGTCAATCTCCTCCATCAT/CCGATCTGACAACTCAAATA	GAAGCGCATCAGCACTCC/CGCCTGCATCCCTTTACC	GCCAAGCCTGAAGAT	4	702/102
1Og2	GAGTCACAGAAAACCAAACC/GTGAAGTCAAAAGCCTCATT	CCTGTACTTGAGACCGACACTCAT/TTCCCCCGGGTGGAGTAT	CTGTCGAGGCATATAGC	4	616/122
1La791	AGCACGTAAAGATGCTGGCT/CCGTCACTCTCGATGCTTGA	TGGACGAGCTTATCAAGTTAACAA/CGCCAGCACAATTTACAACA	ACAGAGTTAAAGGTCGTTAC	2	502/106
1La792	TCGACGTCGATGCAGTTGAA/AAAACCCCCAGAAAATGCGC	GGTAGTACTGCTGACGGTAGTTCG/GAGGGCCTGTTTGTAACGAGA	AACTCCTGCTCCGCC	2	480/109
1La793	AATGGGAGTCAACGAACCGT/CGAAGGATCTCGCCTATCGC	TGCAGCTCAAGGTATCGTATTTCG/TGCAACGGTAGTACTCGGAGTGAT	GTCAGGGCTGAGCCAC	2	407/105
1La794	GCTACGTCATCGACTCCCAG/ACTATGCCCGGTTGCAATCA	ATGGAATACAGAAGTCGGAGAGG/ACGCGGAAAATTCGTTTG	CAGAGAGTACTGATATGCTG	2	510/112
1La795	TGGTAGGTGGTCTCTAGGCC/GCGTATACTCGGCATCCACA	ACATTGCGAGAGGTTTCCA/GACGGACAACGAAGTTTCAGTA	CAGGATATCTGGCTGG	2	451/106
1La796	GAGTTTTGCGAGCGTTGGTT/TGTGCAGGGACACCGATAAC	TTCGAGAAGCCGGTTCGC/TACACCGATGACAGCACCAGTAGA	CACCTGACGACGAAA	2	510/111
2KaLa2	ACATGACCCTCCTTGGTGTC/GAGTCTTCCAACCAGCGAAG	GGTATCATGTCACTGGCTTATGGA/CGACCATATCTTGCCACTCCTG	CAATCAAGAACAACACG	1 + 3	513/97
2La9K2	GCGGTAGTATCGCCATTTGT/TGGGAATCTGAAACCCATGT	GGCCAAGTCCAGCAACAATC/GGGCATTTGTTGAGTTCACGAGT	ACCTACCAGGACACAGA	1 + 2	474/123
3La94K1	ACGGGTGTCATGCCTAGTTC/CGTCATCTCTCCCCAAACTC	ACGCCTGTCTCAACATTTCCTG/GCTCCGCTCTTGATCCAGAA	CCTGCAATCTGACTCG	1 + 2 + 3	481/112
3La94K2	GCGGTAGTATCGCCATTTGT/TGGGAATCTGAAACCCATGT	GGGATCGGTTTCGGGACT/ACAGAAGGCTCGGGAAGCTTA	GCTCGGGAAGCTTAGA	1 + 2 + 3	474/112
3La94K3	TCAGACAAGCTGCAAACACC/CCAAGGGAGAAAGTTGGTCA	CACCAGCATCTGGAAACGTAC/AGCCTCCGAATAATCAACGA	TCTCCTGATGATCCATT	1 + 2 + 3	511/110
3La94K4	CTACGGTCCATCCCTCAGAA/CTTTGAGCTTGCCGAAAATC	GGATGGCTTTCCCAGAGCTAAAC/GCGACGATAGCCCATGATG	CTTGGCTCATGGCCT	1 + 2 + 3	540/122
3La94K5	CCCGGTTATTTCGGTAAGGT/CCTCCTTGATCTTCCGTTCA	CTGAGCAGCGTGACGCCTAC/ATGGGGATCTCGGGAATGC	CGGGAATGCGGCCCT	1 + 2 + 3	472/118
3La94K6	TGCTTCCATTGTGCATTGTT/TTTTAGTGGCCTTCCACAGC	TTGGGTTGGAAGACTAAGATTCCT/TATGACGCCATTCTTAACGTCGA	GACTTATTCAGCAATGTCTC	1 + 2 + 3	541/132

# *Numbers preceding letters in Assay names indicate the number of A. flavus isolates targeted by the assays. Where there is more than one assay per A. flavus isolate, letters in the assay name are followed by the serial number of the assay*.

* *1 = Ka16127, 2 = La3279, 3 = La3304, 4 = Og0222, 1 + 2 = Ka16127 + La3279, 1 + 3 = Ka16127 + La3304, 1 + 2 + 3 = Ka16127 + La3279 + La3304*.

§ *Outer amplicon size/inner amplicon size*.

### Pyrosequencing Assay Refinement

Quality of pyrosequencing assays was checked initially with PyroMark Q24 v2.0.7 software, after which the assays were refined by assaying each target DNA after serial dilution with DNA from the non-target *A. flavus* genotype AF13. Target DNAs were tested at five percentages (100:0, 75:25, 50:50, 25:75, and 0:100). The first and the last treatments served as positive and negative controls, respectively. Proportions of target alleles quantified by pyrosequencing assays at each dilution were fitted into polynomial regression models and compared with actual proportions of target DNA. Each experiment was performed twice, with a completely randomized design and three replications. Goodness of fit for each assay model was determined using the coefficient of determination for the model. Data were subjected to ANOVA and regression models in SAS v9.2 (SAS Institute Inc., Cary, NC).

### Use of Pyrosequencing to Determine Successful Application

Validation of biocontrol application was required to meet contractual requirements and to provide a scientific basis for performance payments intended only for maize confirmed to have been treated. Collection and sampling of maize was previously described (Bandyopadhyay et al., 2019). Briefly, maize from groups of farmers were aggregated by middle men that assisted with training, distribution of the biocontrol product, and marketing of the harvested grain. Thirty kilogram composite samples were taken from ~30 ton lots of aggregated maize by randomly sampling 100 g of maize from each of 300 bags. A 5 kg subsample was taken from each 30 kg sample after homogenization. Subsamples were transported to IITA, Ibadan, Nigeria, where they were milled, homogenized and stored until use.

DNA extracted from maize harvested from fields purported to have been treated were analyzed for the proportion of the *A. flavus* in the maize samples composed of the active ingredients. Proportions were determined using genotype-specific and multi-genotype pyrosequencing assays. Two hundred and ninety-two (292) pelleted DNA samples extracted from the ground maize subsamples were analyzed. DNA extraction was performed at the International Institute of Tropical Agriculture (IITA), Ibadan, Nigeria, following protocols described above, and shipped to the USDA-ARS Lab in Tucson, AZ for pyrosequencing analyses. In Tucson, the DNA samples were re-suspended in 100 μl of purified, autoclaved water, vortexed for 10 s and centrifuged at 14,000 × g for 5 min. Thereafter, 50 μl aliquots of the supernatant were transferred to new 1.5 ml centrifuge tubes as working sub-samples. The remainder of the diluted DNA was stored at −20°C.

To compensate for DNA of reduced quality, 5–10 μl of template DNA was used for both outer and inner amplifications (dependent on ability to visualize PCR products in agarose gel). Outer PCR products were used as template for the inner reaction, after reacting with ExoSAP-IT (Thermo Fisher Scientific Inc., Waltham, MA) to remove unincorporated nucleotides. Biotinylated amplicons from the inner amplifications served as templates for quantitative pyrosequencing, regardless of amplicon quality.

A three-step approach was used to validate application. First, all 292 samples were assayed using 3La94K1, a multi-genotype assay targeting a combination of Ka16127 + La3279 + La3304. Samples with ≥70% of the target active ingredient genotypes were passed, and no further processing of the passed samples was done. Samples with frequencies of the target alleles below 70% were assayed with 1Og2 targeting Og0222. If the sum of Og0222 and Ka16127 + La3279 + La3304 (from 3Ka94K1) was ≥70%, the samples were passed, and no further assays were done on the passed samples. Samples with <70% of the target genotypes, after processing with 1Og2 were analyzed with the second three-isolate assay (3La94K2), and the output added to that 1Og2. If the sum of output from both assays was ≥70%, the samples were passed, otherwise they were deemed to have failed the validation test.

## Results

### Pyrosequencing Assay Refinement

**Figure 1:**
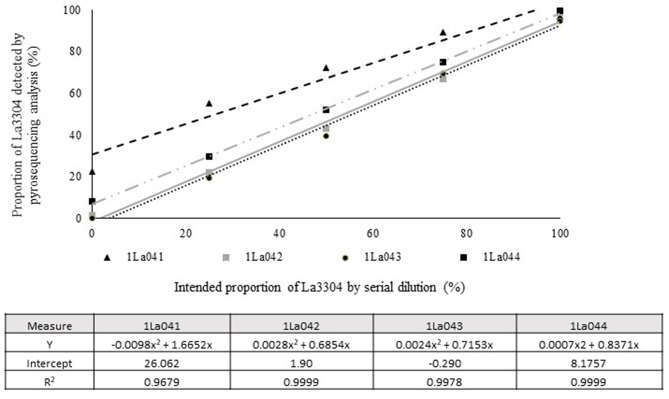
Intended proportion of Aflasafe active ingredient La3304 mixed in varying proportions with *A. flavus* AF13 DNA vs. frequency of La3304 detected using pyrosequencing assays 1La041, 1La042, 1La043, and 1La044.

**Figure 2:**
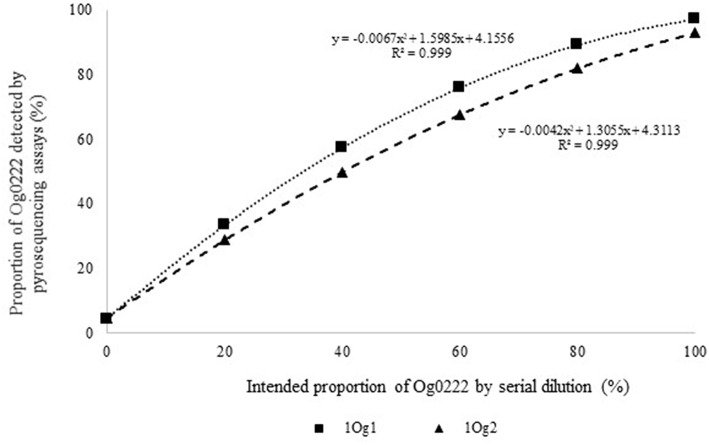
Intended proportion of Aflasafe active ingredient Og0222 DNA mixed in varying proportions with *A. flavus* isolate AF13 vs. frequency of Og0222 detected using pyrosequencing assays 1Og1 and 1Og2.

**Figure 3:**
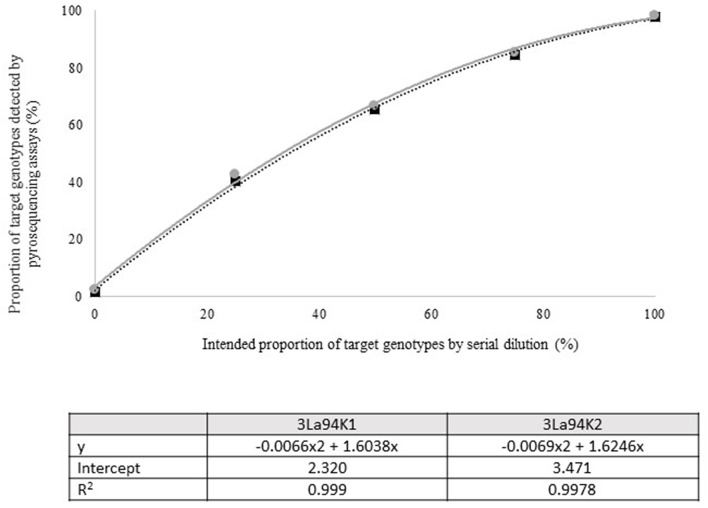
Intended proportion of Aflasafe active ingredients Ka16127, La3279, and La3304, mixed in varying proportions with *A. flavus* isolate AF13 vs. frequency of Ka16127 + La3279 + La3304 detected using pyrosequencing assays 3La94K1 and 3La94K2.

Quantitative pyrosequencing assays were designed targeting SNPs identified with the outlined bioinformatic approach and residing in a variety of regions of the genomes of the active ingredients. Although, all the developed assays were at least partially effective at quantifying frequencies of the target SNPs in pools of DNA, characteristics of the response curves differed. Linear curves from regression models for the two Ka16127-specific single-genotype assays showed an excellent linear relationship (*R*^2^ = 0.9998) between the proportion of target DNA detected by the assays in serial dilutions with AF13 DNA and the intended proportion. Relationships were consistent across all levels of the serial dilution (Table 3). Analysis of data from the six La3279-specific pyrosequencing assays revealed either linear or polynomial relationships to be optimal between proportion of the target DNA detected by the pyrosequencing assays and the intended proportion. Coefficients of determination (R^2^ values) for all models were excellent (*R*^2^ > 0.9), suggesting useful predictive value. However, regression curves intercepted the ordinate axis between 6.3 and 56.0. The most useful assay across the range of target DNA was 1La795 (Table 3). Similarly, the four La3304-specific assays produced response curves with excellent R^2^ values (*R*^2^ > 0.9) and good predictive value across the assayed percentages of target DNA, but with considerable variation in the Y intercept (Figure 1). The two Og0222-specific assays had excellent coefficients of determination (*R*^2^ > 0.9) (Figure 2). All the multi-genotype pyrosequencing assays similarly had excellent coefficients of determination (*R*^2^ > 0.9) with polynomial regression models, indicating that most variance in the data was accounted for by the models (Figure 3). The two assays selected to monitor treatment of maize in Nigeria had interception points between two and four on the ordinate axis with excellent curve fit across all the serial dilution range (Figure 3). Output from single-genotype assays, when summed and compared with multi-isolate assays for the target isolates showed high degrees of similarity by *t*-tests at *P* < 0.05. This suggests similar accuracy and sensitivity between multi-isolate and single isolate assays.

**Table 3:** Refinement of single-isolate quantitative pyrosequencing assays using mixtures of target and non-target *Aspergillus flavus* isolates.

**Percent of target DNA**	**Percent of target DNA detected by the quantitative pyrosequencing assay**								
	**1Ka1**	**1Ka2**	**1La791**	**1La792**	**1La793**	**1La794**	**1La795**	**1La796**	**1La041**
100	100.00	99.83	99.78	97.87	94.71	100.00	97.92	99.46	96.77
75	79.10	77.50	88.01	82.38	82.38	85.92	81.58	98.92	89.43
50	56.14	54.17	60.83	67.26	60.83	67.26	61.95	80.94	72.41
25	34.17	30.84	48.61	42.32	39.28	48.61	42.32	62.97	55.39
0	12.21	7.50	22.69	1.60	7.44	6.26	1.42	10.34	22.46
AF36	ND	ND	5.18	1.18	0.65	1.65	0.59	8.60	ND
SS19-14	9.92	2.13	1.56	0.99	0.00	0.00	0.00	7.93	0.78
MS14-19	0.00	0.00	ND	ND	ND	ND	ND	ND	0.00
Water	0.00	0.00	0.00	0.00	0.00	0.00	0.00	0.00	0.00
Critical value of studentized range	5.304	5.304	5.304	5.304	5.304	5.304	5.304	5.304	5.304
Minimum significant difference	5.523	4.709	7.251	12.662	70.096	6.926	11.725	4.000	57.287

*ND, not determined*.

**Figure 4:**
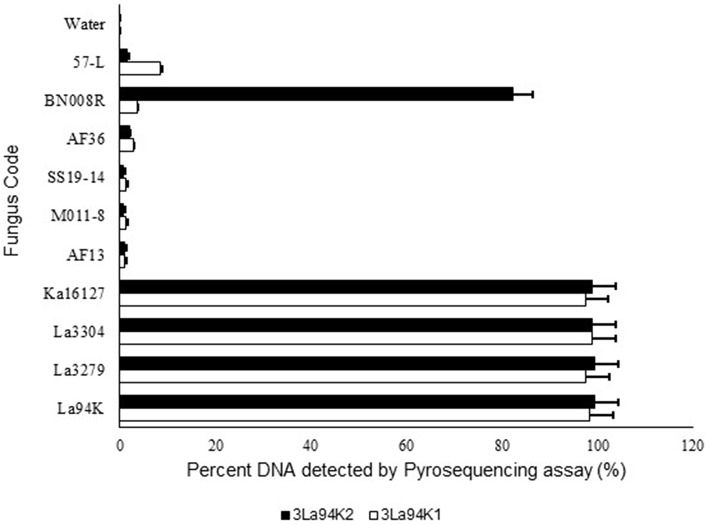
Response of pyrosequencing assays designed to simultaneously detect three active ingredients (Ka16127, La3304, La3279) of Aflasafe to DNA from the three targeted active ingredients and several related *Aspergillus* section *Flavi* fungi.

The two selected multi-isolate assays, 3La94K1, and 3La94K2, were equally effective at detecting either any of the three targeted active ingredients (La3304, La3279, and Ka16127) or mixtures of the three (Figure 4). Furthermore, the assay had low sensitivity to five other *A. flavus* L morphotypes genotypes (57-L, AF36, SS19-14, AF13, and MO11-8). However, assay 3LA94K2 was sensitive to *A. aflatoxiformans* isolate BN008R (Singh and Cotty, 2018; Frisvad et al., 2019) providing a response significantly higher than the baseline. Assay 3LA94K1 was not sensitive to BN008R (Figure 4).

### Analysis of Samples With Quantitative Pyrosequencing Assays

Quantitative pyrosequencing assays specific for the active ingredients detected and quantified the active ingredient genotypes associated with maize samples. Of the 292 samples assayed, 172 (59%) passed the validation tests with either assay 3La94K1 or assay 3La94K2 alone. These two assays quantify three of the four active ingredients. The number of passed samples increased to 274 (94%) when results from assay 1Og2, which quantifies the fourth active ingredient, were added. Eighteen (18) samples (6.2%) were considered to be maize from fields that were not treated properly because the pyrosequencing assays indicated that the four active ingredients composed <70% of the *A. flavus* associated with the maize.

### DNA Extraction From Ground and Whole Maize

The DNA extraction protocols resulted in DNA adequate for the developed pyrosequencing assays. Washing both whole and ground maize samples with 0.1% TWEEN®80 resulted in up to a 20% increase in the frequency of assays which passed the PyroMark Q24 internal quality controls on the first attempt.

## Discussion

Biocontrol products with atoxigenic *A. flavus* active ingredients are inexpensive effective tools farmers use to reduce crop aflatoxin content (Cotty and Bayman, 1993; Mehl et al., 2012; Atehnkeng et al., 2014; Bandyopadhyay et al., 2016; Abbas et al., 2017). Treatments are effective at reducing the likelihood that crops have unacceptable aflatoxin content. Industries benefit from use of atoxigenics because these products reduce risk associated with the highly heterogeneous nature of contamination, year to year fluctuations in aflatoxin incidences, and impacts of weather events on severity of contamination (Cotty and Jaime-Garcia, 2007; Medina et al., 2014; Bandyopadhyay et al., 2016). For this reason, some purchasers of crops, including processors, dairies, flour mills and market development projects require or recommend that crops brought to them be treated with an atoxigenic *A. flavus* biocontrol product during crop development. In some cases, biocontrol treatments are required to ensure long-term benefits and to make continuing improvements to the aflatoxin vulnerability in areas from which end-users traditionally draw crops. Also, end-users may seek other advantages, including post-harvest protection during silage operations, storage, and animal feeding operations (Cotty and Mellon, 2006; Prandini et al., 2009; Wu and Khlangwiset, 2010; Alonso et al., 2013; Bandyopadhyay et al., 2016).

Although on average, crops treated with atoxigenic biocontrol products have less aflatoxins than untreated crops, aflatoxin contamination is highly variable among fields and, as a result, aflatoxin content alone cannot be used to indicate treatment. Indeed, there are no rapid visual or chemical assays to indicate a crop was properly treated with an atoxigenic biocontrol product. One approach is to isolate individual *A. flavus* from crops, and to characterize each by either VCA (Cotty and Bayman, 1993; Cotty et al., 1994; Ehrlich and Cotty, 2004), or DNA fingerprinting (Grubisha and Cotty, 2015; Islam et al., 2018). However, such methods have significant sampling errors and require trade-off between costs associated with the number of individuals assayed and the desired accuracy. The approach described here allows detection of genetic variants in pools of DNA from millions of individuals, reducing costs and sampling errors associated with culturing and characterizing individuals. Quantitative pyrosequencing is particularly well-suited to determine the percent of the *A. flavus* community containing a target SNP (Das et al., 2008) and allows resolution of small differences not achievable with other methods (Sogin et al., 2006; Siqueira et al., 2012; Harrington et al., 2013; Zhou and Mehl, 2019). In the current study, quantitative pyrosequencing proved a very useful tool for rapidly determining farmer compliance in Nigeria with a market that requires application of a biocontrol product. In this case, the market is one created by the AgResults Initiative (AgResults, 2019), a large multilateral endeavor that uses a pay-for-results model to incentivize private sector adoption of innovative solutions to problems of smallholder farmers. The Nigeria project is the first time such incentivization has been applied to adoption of a plant disease biocontrol product.

In Nigeria, most farmers are small holders with <2 ha planting area and poor yield of <2 tons/ha. This results in single farm total crop value insufficient to support costs of proper crop sampling, sample preparation and aflatoxin analyses. If tests detect unacceptable aflatoxin levels, the farmer has few options to recover both costs of analyses and crop value. For many farmers, the cost of using an atoxigenic strain-based product is less than the cost of performing per field aflatoxin analyses and treatments are invariably associated with reduced aflatoxins (Bandyopadhyay et al., 2016). Low costs of atoxigenic strain-based biocontrol products give small holder farmers a practical alternative to reduce aflatoxin exposure (Ayedun et al., 2017; Johnson et al., 2018, 2019). Proper treatments result in atoxigenic strain active ingredients composing >80% of the crop-associated *A. flavus* population. High frequencies of the atoxigenic-strain active ingredients on a crop is the most reliable indicator of proper treatment (Cotty and Bayman, 1993; Atehnkeng et al., 2014).

From 2014 to 2018, assays described in the current report were used to determine presence of atoxigenic-strain active ingredients on harvested maize and, in so doing, verify proper use of biocontrol by participating farmers. In total 4,288 maize samples from 48,513 farmers who treated 61,645 ha with biocontrol were analyzed with 91% having sufficient incidences of active ingredients to confirm proper use. Verification of proper use resulted in a performance payment (AgResults, 2019).

Quantitative pyrosequencing is highly precise, accurate, and rapid (Mehl and Cotty, 2010, 2013). However, this technology has been underutilized in plant pathology and only recently has been applied to monitoring frequencies of resistance to fungicides (Zhou and Mehl, 2019). Single-genotype pyrosequencing assays provide accurate and rapid quantification of target *A. flavus* genotypes in crop associated populations (Das et al., 2008; Mehl and Cotty, 2011). The current study utilized whole genome analyses to design twenty-four quantitative pyrosequencing assays developed for rapid and simultaneous quantification of multiple *A. flavus* genotypes in maize associated fungal populations. The use of whole genome sequence analyses in the current study also allowed development of assays for simultaneous quantification of multiple genotypes. Use of pyrosequencing with assays similar to those developed here may allow long-term monitoring of *A. flavus* populations and associated design of low cost, area-wide programs to prevent dangerous concentrations of aflatoxins. This results from characteristics of this technology to precisely quantify frequencies of DNA sequence variation in complex microbial populations (Ronaghi, 2001; Mehl and Cotty, 2010; Siqueira et al., 2012). The high throughput and relatively low cost of the pyrosequencing method provides adequate sampling depth to facilitate detection of both dominant and rare individuals within complex microbial populations (Sogin et al., 2006; Kunin et al., 2010; Siqueira et al., 2012), making it also suitable for ecological studies on environmental influences to *A. flavus* population structure. However, no technology is sufficiently inexpensive to allow frequent economic monitoring of individual small holder crops. Therefore, application of this and similar technologies will likely rely on composite samples from multiple fields as performed in the current study to determine carryover, dispersal, and long-term efficacy of management programs.

Previous studies (Das et al., 2008; Mehl and Cotty, 2010) developed assays for known SNPs in a few specific genes. Whole genomes were scanned in the current study for useful SNPs. The utilized whole genome searches were not able to find SNPs shared by all four target isolates because of divergence of *A. flavus* Og0222 from Ka16127, La3279, and La3304. However, the assays developed for targeting the three latter fungi demonstrate the concept of simultaneous monitoring of multiple genotypes with a single assay. With the multi-genotype assays, a single instrument could simultaneously quantify the target genotypes from up to 240 crop samples per day after DNA extraction, an endeavor that would take several months and greatly increase labor with VCA (Mehl and Cotty, 2010). As increasing numbers of genomes of closely related fungi become available, and sufficient computing power is widely distributed, similar pyrosequencing assays may be developed to monitor incidences of any genotype independent of mutations that influence phenotype. As in the current study, nesting of PCR can be applied to increase specificity and yield of rare genotypes independent of the phenotypes of possible adaptive significance. Indeed specificity of assays in the current study is derived from five primers: Two for the outer PCR; two for the inner PCR, and the primer used to initiate the sequence by synthesis reaction during pryrosequencing. Such specific assays may allow dissection of population genetics and dispersal independent of selected for adaptations.

In the current study, pyrosequencing assays targeting the same Aflasafe active ingredients differed in sensitivity and accuracy. The observed differences were probably the result of variation in regions flanking the SNP. Actual frequencies of SNPs in the assayed populations and presence in non-target genotypes could also affect ability of assays to discriminate between targets and non-targets. The current study used non-target genotypes to identify useful SNPs. However, it was considered prudent to call multiple SNPs for each Aflasafe genotype, and to test several multiple-genotype assays to select the best-performing assays for deployment. The prudence of this approach was affirmed by observed variation in performance of pyrosequencing assays targeting the same Aflasafe active ingredient genotype (Figure 1). All developed assays readily detected variation in incidence of the targeted active ingredients. However, preferred assays allowed detection that most closely approximated linear response curves, had the highest coefficient of determination (R^2^), and a Y intercept most approximate to zero (Figure 1). The DNA extraction protocols and standardized nested PCR and pyrosequencing methods in the current work can be used for assays for targets beyond those examined here.

## Conclusion

Pyrosequencing assays provide a flexible and robust tool for assessment of efficacy of biocontrol technologies directed at altering the *A. flavus* community structure. These assays can be used to confirm proper use of biocontrol products in a timeframe of potential value to commercial agriculture. Rapid simultaneous monitoring of multiple genotypes in complex crop-associated *A. flavus* populations may be useful for monitoring the environmental fate of active ingredients and cumulative benefits accrued from varying patterns of biocontrol product use.

## Data Availability Statement

The whole genome *Aspergillus flavus* sequence data analyzed in this study and unique to this study are deposited at DDBJ/ENA/GenBank under BioProject PRJNA565924, SUBID SUB6303482, accession VYXL00000000 for Ka16127, VYXK00000000 for La3279, VYXJ00000000 for La3304, and VYXI00000000 for Og0222.

## Author Contributions

RB and PC designed the overall project and secured funds for the studies from which the data are derived. BA performed the whole genome analyses. AA organized and supervised the farmer training and sampling of the crops. KS, BA, KC, RB, and PC collaborated on designing the analysis pipeline. KS, AA, JA, AO-B, and PK performed the sample preparation, DNA isolation, and pyrosequencing. KS analyzed the data and drafted the original manuscript. PC and RB edited the manuscript. All authors read, reviewed, and approved the final manuscript.

### Conflict of Interest

The authors receive no direct financial benefit from the marketing of the biocontrol technology described in this work. Initial patents for use of atoxigenic strains to prevent aflatoxin contamination were filed in 1988 and awarded by the US patent office to the US Department of Agriculture in 1992 and 1994 with PC as the inventor. The patent protection has expired. The manufacturing process for and the compositions of Aflasafe, the biocontrol product discussed in the current work, are not patented and are used for several atoxigenic strain-based products that differ primarily in the active ingredient genotypes. In addition to the Nigeria Aflasafe product, these products include other products bearing the Aflasafe name (e.g., Aflasafe SN01), AF36 Prevail in the US, and AF X-1 in Italy (Mauro et al., 2015; Bandyopadhyay et al., 2016; Ortega-Beltran and Bandyopadhyay, 2019). The fungal isolates used as active ingredients of Aflasafe Nigeria are considered a portion of the bioresources of Nigeria and, as such, are not patented. However, atoxigenic genotypes suitable for biocontrol applications have been found in all regions where active ingredients have been sought (Bandyopadhyay et al., 2016). The Aflasafe name is a Trademark of the International Institute of Tropical Agriculture (IITA). During the course of this study, IITA manufactured and marketed Aflasafe. Manufacturing and distribution has been transferred to the private sector during 2019. IITA charges a licensing fee to manufacturers for use of the Aflasafe name and associated technology transfer. RB, AO-B, JA, PK, and AA were employed by IITA. The remaining authors declare that the research was conducted in the absence of any commercial or financial relationships that could be construed as a potential conflict of interest.
